# Triethyl­ammonium bis­(2-oxido-2,2-diphenyl­acetato-κ^2^ *O* ^1^,*O* ^2^)anti­monate(III)

**DOI:** 10.1107/S1600536809054853

**Published:** 2010-01-09

**Authors:** Md. Yeamin Reza, Md. Motahar Hossain, Md. Rabiul Karim, Md. Tofazzal Hossain Tarafder, David L. Hughes

**Affiliations:** a Department of Chemistry, University of Rajshahi, Rajshahi 6205, Bangladesh; b School of Chemistry, University of East Anglia, Norwich NR4 7TJ, England

## Abstract

The coordination around the Sb atom in the title compound, (C_6_H_16_N)[Sb(C_14_H_10_O_3_)_2_], is fourfold in a pseudo-trigonal-bipyramidal pattern in which one of the equatorial sites is occupied by the stereoactive lone pair of electrons. The four ligating atoms comprise the hydoxylate and carboxyl­ate O atoms from two independent benzilate ligands, each of which forms a five-membered chelating ring, spanning an axial and an equatorial site about the Sb atom. The hydroxy­late atoms occupy the two equatorial sites, and the carboxyl­ate atoms are in the pseudo-axial sites; the O—Sb—O angle is 147.72 (5)°. One carboxyl­ate group shows quite different bond lengths from those of the other group; one O atom is clearly the carbonyl atom and the other O atom the hydroxy­late atom. In the other ligand, there is less distinction in the C—O bonds. This is presumably related to the carbonyl O atom being the acceptor atom of a strong N—H⋯O hydrogen bond, which links the ammonium cation to the Sb complex anion.

## Related literature

For metal–carboxyl­ate and alkoxide complexes: Reza *et al.* (1998 ▶, 1999 ▶, 2003 ▶); Tarafder *et al.* (2008 ▶). For anti­mony(III) complexes, see: Razak *et al.* (2002 ▶); Vijjulatha *et al.* (1997 ▶). For α-hydroxy­carboxyl­ate complexes, see: Hartley *et al.* (1991 ▶); Smith *et al.* (1992 ▶, 1993 ▶); Smith & Kennard (1996 ▶); Bott *et al.* (2000 ▶); Redshaw & Elsegood (2007 ▶); Redshaw *et al.* (2005 ▶); Chandrasekhar *et al.* (2005 ▶); Vergopoulos *et al.* (1995 ▶); Carballo *et al.* (2004*a* ▶,*b* ▶).
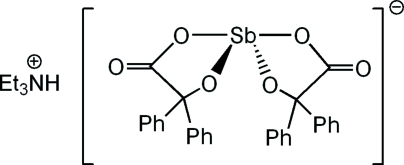


## Experimental

### 

#### Crystal data

(C_6_H_16_N)[Sb(C_14_H_10_O_3_)_2_]
*M* *_r_* = 676.39
Monoclinic, 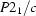

*a* = 12.34379 (13) Å
*b* = 10.63317 (12) Å
*c* = 22.5264 (3) Å
β = 90.6466 (11)°
*V* = 2956.49 (5) Å^3^
*Z* = 4
Mo *K*α radiation
μ = 0.98 mm^−1^
*T* = 140 K
0.28 × 0.27 × 0.09 mm

#### Data collection

Oxford Diffraction Xcalibur 3/CCD diffractometer
Absorption correction: multi-scan (*CrysAlis RED*; Oxford Diffraction, 2008 ▶) *T*_min_ = 0.911, *T*_max_ = 1.000
61564 measured reflections
6772 independent reflections
5645 reflections with *I* > 2σ(*I*)
*R*_int_ = 0.054

#### Refinement

*R*[*F* ^2^ > 2σ(*F* ^2^)] = 0.023
*wR*(*F* ^2^) = 0.049
*S* = 1.01
6772 reflections
383 parameters
H atoms treated by a mixture of independent and constrained refinement
Δρ_max_ = 0.41 e Å^−3^
Δρ_min_ = −0.42 e Å^−3^

### 

Data collection: *CrysAlis CCD* (Oxford Diffraction, 2008 ▶); cell refinement: *CrysAlis RED* (Oxford Diffraction, 2008 ▶); data reduction: *CrysAlis RED*; program(s) used to solve structure: *SHELXS97* (Sheldrick, 2008 ▶); program(s) used to refine structure: *SHELXL97* (Sheldrick, 2008 ▶); molecular graphics: *ORTEPII* (Johnson, 1976 ▶) and *ORTEP-3* (Farrugia, 1997 ▶); software used to prepare material for publication: *SHELXL97* and *WinGX* (Farrugia, 1999 ▶).

## Supplementary Material

Crystal structure: contains datablocks I, global. DOI: 10.1107/S1600536809054853/pb2016sup1.cif

Structure factors: contains datablocks I. DOI: 10.1107/S1600536809054853/pb2016Isup2.hkl

Additional supplementary materials:  crystallographic information; 3D view; checkCIF report

## Figures and Tables

**Table:** 

Sb—O1	2.2091 (12)
Sb—O2	1.9751 (11)
Sb—O4	2.1204 (12)
Sb—O5	1.9662 (11)

**Table:** 

O2—Sb—O1	76.77 (4)
O4—Sb—O1	147.72 (5)
O5—Sb—O1	81.60 (5)
O2—Sb—O4	81.84 (5)
O5—Sb—O2	100.79 (5)
O5—Sb—O4	78.97 (5)

**Table 2:** Hydrogen-bond geometry (Å, °)

*D*—H⋯*A*	*D*—H	H⋯*A*	*D*⋯*A*	*D*—H⋯*A*
N7—H7⋯O11	0.93 (2)	1.78 (2)	2.706 (2)	177.5 (18)

**Fig. 1.**
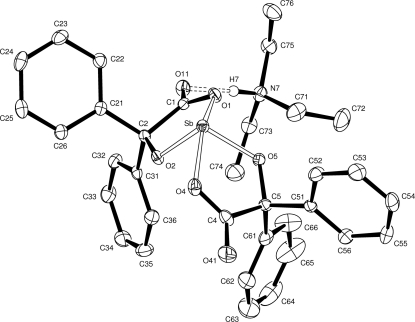
View of a molecule of (Et3NH)[Sb(Ph2COCOO)2] (1), indicating the atom numbering scheme. Hydrogen atoms (except that involved in the hydrogen bond) have been omitted for clarity. Thermal ellipsoids are drawn at the 30% probability level.

**Fig. 2.**
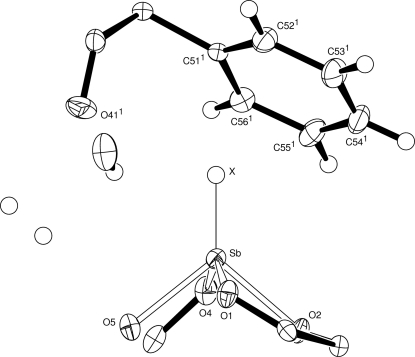
Environs of lone-pair of electrons (around the point X) of the antimony atom. The superscript denotes the symmetry operation: 1 - x, y - 1/2, 1/2 - z.

## supplementary crystallographic information

### Comment

In an ongoing study of metal-carboxylate and -alkoxide complexes (Reza *et al.*, 1998, 1999, 2003; Tarafder *et al.*, 2008), with particular interest in their antimicrobial and catalytic properties, we are exploring the coordination chemistry of some α-hydroxycarboxylate ions which contain both the ligating groups of interest and whose parent acids have important roles in enzyme reactions. Often, the α-hydroxycarboxylate ligand forms a simple chelating ring with a metal (*e.g.* Vergopoulos *et al.*, 1995), but terminal and bridging modes have also been found (*e.g.* Carballo *et al.*, 2004*a*, 2004*b*). The bridging modes, using the three (or more) available oxygen donor atoms, allow the formation of oligomeric complexes, for example the macrocyclic rings formed between benzilate ions and AlMe_3_ (Redshaw *et al.*, 2005) or ZnEt_2_ (Redshaw *et al.*, 2007), and the cages formed by 9-hydroxy-9-fluorenecarboxylic acid with *n*-BuSn(O)OH (Chandrasekhar *et al.*, 2005). We recall also the series of citrate complexes in which the citrate ion chelates an antimony ion and bridges to a range of simple cations (alkali metals, copper, silver, *etc*) to form a variety of polymeric structures (Hartley *et al.*, 1991, Smith *et al.*, 1992, 1993, Bott *et al.*, 2000).

We have now prepared an Sb(III) complex of the benzilate (diphenylglycolate) ion, with Et_3_NH^+^ as the counterion. This cation is non-coordinating and has a single N-H group with potential for formation of hydrogen bonds. The product, the title compound (I), shows (Figure 1) the antimony atom coordinated by two independent benzilate ligands with very similar conformations, each with a five-membered chelating ring which bridges an axial and equatorial site of the pseudo trigonal bipyramidal coordination polyhedron; the fifth, equatorial coordination site is occupied by a stereoactive lone-pair of electrons in the style typical of antimony(III) complexes of citrate and other α-hydrocarboxylate ions. In all these Sb(III) complexes, the alkoxide O atom is in an equatorial position and one of the carboxylate O atoms is in an axial site, and the axial Sb—O bonds are slightly longer than the equatorial bonds. Slight differences in the dimensions of the carboxylate groups in our complex are thought to result from the participation of one of these groups as acceptor of a good, almost linear hydrogen bond, N(7)—H(7)···O(11), from the Et_3_NH^+^ cation. The Sb(OOCCOPh_2_)_2_^-^ anion and Et_3_NH^+^ cation thus appear as a discrete ion-pair unit in the crystal.

On the Sb atom, the lone-pair of electrons (and vacant coordination site) are bounded by the phenyl group of C(51^1^-56^1^) and O(41^1^) of the molecule at (1 - *x*, *y* - 1/2, 1/2 - *z*), Figure 2; a point *X* calculated along the bisecting vectors towards the coordination site, at 1.5 Å from the Sb atom, has *X*···C distances in the range 2.45 - 2.90 Å and *X*···O(41^1^) 2.70 Å.

There are two intermolecular 'weak hydrogen bonds', *viz* C(24)—H(24)···O(2^2^) and C(75)—H(75b)···O(41^3^) with H···O contacts of 2.44 and 2.49 Å. The other major intermolecular contacts are C—H···π interactions, *e.g.* the phenyl group of C(21–26) is bounded on one side by the hydrogen atom H(54^1^), and on the other side H(73*b*^4^) where these hydrogen atoms are displaced 2.95 and 2.61 Å from the ring mean-plane. The ring of C(31–36) has a similar interaction, with H(74*b*) displaced 2.73 Å from the ring plane, but on the opposite side, H(54^5^) is directed at the C(35)—C(36) bond. As noted above, the ring of C(51–56) has a lone-pair of electrons on one side; H(76*a*^6^) is on the opposite side but points more to the C(51) end of the ring. The hydrogen atoms H(35 and 36) and H(72*a*^7^), approaching the ring of C(61–66), are also directed at the edges of the ring. [Symmetry operations, denoted by superscripts are: 1: 1 - *x*, *y* - 1/2, 1/2 - *z*; 2: -*x*, *y* - 1/2, 1/2 - *z*; 3: *x*, 3/2 - *y*, 1/2 + *z*; 4: -*x*, 1 - *y*, 1 - *z*; 5: *x* - 1, *y*, *z*; 6: 1 - *x*, 1 - *y*, 1 - *z*; 7: 1 - *x*, 2 - *y*, 1 - *z*].

Molecular dimensions are available in the archived CIF.

### Experimental

The title complex, (Et_3_NH) [Sb(Ph_2_COCOO)_2_] (1) was synthesized by adding, with stirring, a mixture of benzilic acid (2 mmol) and triethylamine (2 mmol) in acetonitrile (50 ml) to 30 ml of an aqueous solution of SbCl_3_ (1 mmol). The stirring was continued for another 30 min at 60 C. Precipitates initially formed were filtered and the filtrate was concentrated to one-third of its original volume (*ca* 30 ml). The colourless single crystals of 1 which appeared after a week were collected, washed with water and dried in air at room temperature, Mp. 558 K.

### Refinement

The non-hydrogen atoms were refined with anisotropic thermal parameters. The ammonium hydrogen atom, H(7), was located in a difference map and was refined freely; the remaining hydrogen atoms were included in idealized positions and their *U*_iso_ values were set to ride on the *U*_eq_ values of the parent carbon atoms. The largest difference peak (near Sb) and hole were 0.41 and -0.42 e.Å^-3^.

### Crystal data

**Table:**

(C_6_H_16_N)[Sb(C_14_H_10_O_3_)_2_]	*F*(000) = 1384
*M**_r_* = 676.39	*D*_x_ = 1.520 Mg m^−^^3^
Monoclinic, *P*2_1_/*c*	Mo *K*α radiation, λ = 0.71073 Å
*a* = 12.34379 (13) Å	Cell parameters from 33278 reflections
*b* = 10.63317 (12) Å	θ = 3.1–32.3°
*c* = 22.5264 (3) Å	µ = 0.98 mm^−^^1^
β = 90.6466 (11)°	*T* = 140 K
*V* = 2956.49 (5) Å^3^	Plate, colourless
*Z* = 4	0.28 × 0.27 × 0.09 mm

### Data collection

**Table:**

Oxford Diffraction Xcalibur 3/CCD diffractometer	6772 independent reflections
Radiation source: Enhance (Mo) X-ray Source	5645 reflections with *I* > 2σ(*I*)
graphite	*R*_int_ = 0.054
Detector resolution: 16.0050 pixels mm^-1^	θ_max_ = 27.5°, θ_min_ = 3.1°
Thin–slice φ and ω scans	*h* = −16→16
Absorption correction: multi-scan (*CrysAlis RED*; Oxford Diffraction, 2008)	*k* = −13→13
*T*_min_ = 0.911, *T*_max_ = 1.000	*l* = −29→29
61564 measured reflections	

### Refinement

**Table:**

Refinement on *F*^2^	Primary atom site location: structure-invariant direct methods
Least-squares matrix: full	Secondary atom site location: difference Fourier map
*R*[*F*^2^ > 2σ(*F*^2^)] = 0.023	Hydrogen site location: inferred from neighbouring sites
*wR*(*F*^2^) = 0.049	H atoms treated by a mixture of independent and constrained refinement
*S* = 1.01	*w* = 1/[σ^2^(*F*_o_^2^) + (0.0274*P*)^2^] where *P* = (*F*_o_^2^ + 2*F*_c_^2^)/3
6772 reflections	(Δ/σ)_max_ = 0.003
383 parameters	Δρ_max_ = 0.41 e Å^−^^3^
0 restraints	Δρ_min_ = −0.42 e Å^−^^3^

### Special details

**Table:**

Experimental. CrysAlisPro RED, Oxford Diffraction Ltd., Version 1.171.32.24 Empirical absorption correction using spherical harmonics, implemented in SCALE3 ABSPACK scaling algorithm.
Geometry. All s.u.'s (except the s.u. in the dihedral angle between two l.s. planes) are estimated using the full covariance matrix. The cell s.u.'s are taken into account individually in the estimation of s.u.'s in distances, angles and torsion angles; correlations between s.u.'s in cell parameters are only used when they are defined by crystal symmetry. An approximate (isotropic) treatment of cell s.u.'s is used for estimating s.u.'s involving l.s. planes.
Refinement. Refinement of *F*^2^ against ALL reflections. The weighted *R*-factor *wR* and goodness of fit *S* are based on *F*^2^, conventional *R*-factors *R* are based on *F*, with *F* set to zero for negative *F*^2^. The threshold expression of *F*^2^ > 2σ(*F*^2^) is used only for calculating *R*-factors(gt) *etc*. and is not relevant to the choice of reflections for refinement. *R*-factors based on *F*^2^ are statistically about twice as large as those based on *F*, and *R*- factors based on ALL data will be even larger.

### Fractional atomic coordinates and isotropic or equivalent isotropic displacement parameters (Å^2^)

**Table:**

	*x*	*y*	*z*	*U*_iso_*/*U*_eq_	
Sb	0.344289 (9)	0.578178 (10)	0.306991 (5)	0.01409 (4)	
O1	0.31229 (9)	0.51859 (11)	0.39909 (6)	0.0186 (3)	
C1	0.21403 (13)	0.53115 (16)	0.41481 (8)	0.0146 (3)	
O11	0.17823 (10)	0.49894 (12)	0.46352 (5)	0.0199 (3)	
C2	0.13642 (13)	0.58983 (15)	0.36784 (7)	0.0125 (3)	
O2	0.19745 (9)	0.64426 (11)	0.32163 (5)	0.0144 (2)	
C21	0.06322 (13)	0.48800 (16)	0.34061 (8)	0.0137 (3)	
C22	0.07864 (14)	0.36064 (17)	0.35079 (8)	0.0187 (4)	
H22	0.1310	0.3342	0.3781	0.022*	
C23	0.01619 (15)	0.27202 (17)	0.32035 (8)	0.0225 (4)	
H23	0.0279	0.1867	0.3270	0.027*	
C24	−0.06292 (14)	0.30980 (18)	0.28049 (8)	0.0231 (4)	
H24	−0.1044	0.2505	0.2601	0.028*	
C25	−0.07987 (14)	0.43711 (18)	0.27118 (8)	0.0226 (4)	
H25	−0.1336	0.4634	0.2447	0.027*	
C26	−0.01765 (14)	0.52544 (17)	0.30094 (8)	0.0178 (4)	
H26	−0.0300	0.6106	0.2944	0.021*	
C31	0.06915 (13)	0.69218 (15)	0.39781 (8)	0.0142 (3)	
C32	−0.01336 (14)	0.65910 (17)	0.43647 (8)	0.0184 (4)	
H32	−0.0279	0.5746	0.4436	0.022*	
C33	−0.07398 (14)	0.75074 (18)	0.46439 (9)	0.0229 (4)	
H33	−0.1288	0.7277	0.4902	0.027*	
C34	−0.05292 (15)	0.87695 (18)	0.45388 (9)	0.0243 (4)	
H34	−0.0941	0.9388	0.4721	0.029*	
C35	0.02932 (16)	0.90969 (17)	0.41627 (9)	0.0242 (4)	
H35	0.0440	0.9942	0.4095	0.029*	
C36	0.09080 (14)	0.81839 (16)	0.38823 (8)	0.0194 (4)	
H36	0.1464	0.8419	0.3630	0.023*	
O4	0.34473 (9)	0.72582 (12)	0.24372 (5)	0.0201 (3)	
C4	0.39510 (14)	0.82763 (17)	0.25927 (8)	0.0195 (4)	
O41	0.40424 (11)	0.91988 (13)	0.22763 (6)	0.0311 (3)	
C5	0.44681 (13)	0.82505 (16)	0.32260 (8)	0.0153 (3)	
O5	0.43343 (9)	0.70446 (10)	0.34853 (5)	0.0166 (3)	
C51	0.56859 (13)	0.84740 (16)	0.31541 (8)	0.0147 (3)	
C52	0.62838 (14)	0.75725 (17)	0.28514 (9)	0.0220 (4)	
H52	0.5933	0.6876	0.2689	0.026*	
C53	0.73872 (15)	0.76966 (18)	0.27882 (9)	0.0264 (4)	
H53	0.7774	0.7085	0.2585	0.032*	
C54	0.79229 (15)	0.87246 (19)	0.30243 (9)	0.0259 (4)	
H54	0.8669	0.8803	0.2986	0.031*	
C55	0.73385 (15)	0.96320 (19)	0.33175 (9)	0.0249 (4)	
H55	0.7693	1.0330	0.3475	0.030*	
C56	0.62251 (14)	0.95149 (16)	0.33797 (8)	0.0197 (4)	
H56	0.5839	1.0140	0.3574	0.024*	
C61	0.39459 (13)	0.92205 (17)	0.36336 (9)	0.0210 (4)	
C62	0.33837 (16)	1.0251 (2)	0.34307 (11)	0.0354 (5)	
H62	0.3300	1.0384	0.3025	0.043*	
C63	0.2942 (2)	1.1093 (2)	0.38288 (15)	0.0536 (8)	
H63	0.2562	1.1787	0.3687	0.064*	
C64	0.30550 (18)	1.0921 (2)	0.44250 (14)	0.0515 (8)	
H64	0.2745	1.1487	0.4688	0.062*	
C65	0.3631 (2)	0.9904 (2)	0.46345 (12)	0.0501 (7)	
H65	0.3726	0.9787	0.5041	0.060*	
C66	0.40684 (19)	0.9058 (2)	0.42384 (10)	0.0377 (5)	
H66	0.4452	0.8367	0.4382	0.045*	
N7	0.28608 (12)	0.57191 (14)	0.56292 (7)	0.0187 (3)	
C71	0.38735 (15)	0.6338 (2)	0.54177 (10)	0.0293 (5)	
H71A	0.3677	0.7009	0.5145	0.035*	
H71B	0.4295	0.5727	0.5199	0.035*	
C72	0.45699 (17)	0.6876 (2)	0.59098 (11)	0.0404 (6)	
H72A	0.5203	0.7258	0.5744	0.061*	
H72B	0.4785	0.6215	0.6176	0.061*	
H72C	0.4166	0.7498	0.6123	0.061*	
C73	0.21227 (15)	0.65915 (17)	0.59532 (8)	0.0217 (4)	
H73A	0.2481	0.6871	0.6315	0.026*	
H73B	0.1474	0.6137	0.6064	0.026*	
C74	0.18027 (17)	0.77240 (19)	0.55908 (9)	0.0316 (5)	
H74A	0.1336	0.8253	0.5820	0.047*	
H74B	0.1428	0.7456	0.5237	0.047*	
H74C	0.2440	0.8185	0.5484	0.047*	
C75	0.30507 (16)	0.45419 (17)	0.59820 (8)	0.0234 (4)	
H75A	0.2360	0.4205	0.6107	0.028*	
H75B	0.3472	0.4743	0.6335	0.028*	
C76	0.36437 (17)	0.35545 (19)	0.56253 (9)	0.0313 (5)	
H76A	0.3595	0.2757	0.5823	0.047*	
H76B	0.4391	0.3789	0.5590	0.047*	
H76C	0.3321	0.3492	0.5237	0.047*	
H7	0.2489 (16)	0.5445 (18)	0.5292 (9)	0.021 (5)*	

### Atomic displacement parameters (Å^2^)

**Table:**

	*U*^11^	*U*^22^	*U*^33^	*U*^12^	*U*^13^	*U*^23^
Sb	0.01236 (6)	0.01524 (6)	0.01468 (6)	0.00074 (5)	0.00095 (4)	−0.00073 (5)
O1	0.0142 (6)	0.0211 (6)	0.0203 (7)	0.0006 (5)	−0.0024 (5)	0.0061 (5)
C1	0.0163 (8)	0.0120 (8)	0.0155 (9)	−0.0021 (6)	−0.0032 (7)	0.0003 (7)
O11	0.0208 (6)	0.0252 (7)	0.0137 (7)	−0.0039 (5)	−0.0014 (5)	0.0044 (5)
C2	0.0122 (7)	0.0143 (8)	0.0111 (8)	−0.0004 (6)	0.0001 (6)	0.0017 (7)
O2	0.0104 (5)	0.0199 (6)	0.0128 (6)	0.0004 (5)	0.0016 (5)	0.0050 (5)
C21	0.0116 (8)	0.0173 (9)	0.0122 (8)	−0.0007 (6)	0.0028 (7)	−0.0031 (7)
C22	0.0165 (9)	0.0201 (9)	0.0196 (10)	0.0017 (7)	−0.0009 (7)	−0.0030 (7)
C23	0.0234 (9)	0.0167 (9)	0.0276 (11)	−0.0008 (7)	0.0035 (8)	−0.0064 (8)
C24	0.0186 (9)	0.0298 (11)	0.0211 (10)	−0.0059 (8)	0.0026 (8)	−0.0116 (8)
C25	0.0169 (9)	0.0321 (11)	0.0186 (9)	0.0000 (8)	−0.0040 (7)	−0.0032 (8)
C26	0.0172 (9)	0.0198 (9)	0.0162 (9)	−0.0003 (7)	−0.0013 (7)	−0.0009 (7)
C31	0.0128 (8)	0.0176 (9)	0.0122 (8)	0.0002 (6)	−0.0034 (7)	−0.0017 (7)
C32	0.0169 (9)	0.0177 (9)	0.0208 (10)	−0.0029 (7)	0.0011 (7)	−0.0017 (7)
C33	0.0174 (9)	0.0273 (10)	0.0240 (10)	−0.0012 (8)	0.0046 (8)	−0.0047 (8)
C34	0.0238 (10)	0.0230 (9)	0.0262 (11)	0.0064 (8)	−0.0002 (8)	−0.0079 (8)
C35	0.0302 (10)	0.0151 (9)	0.0274 (10)	0.0010 (8)	−0.0007 (8)	−0.0015 (8)
C36	0.0202 (9)	0.0204 (9)	0.0177 (9)	−0.0018 (7)	0.0004 (7)	0.0015 (7)
O4	0.0179 (6)	0.0271 (7)	0.0153 (6)	−0.0026 (5)	−0.0013 (5)	0.0032 (5)
C4	0.0139 (8)	0.0259 (10)	0.0189 (10)	0.0035 (7)	0.0018 (7)	0.0057 (8)
O41	0.0336 (8)	0.0303 (8)	0.0292 (8)	−0.0038 (6)	−0.0051 (6)	0.0166 (7)
C5	0.0144 (8)	0.0150 (8)	0.0165 (9)	−0.0006 (6)	0.0009 (7)	0.0044 (7)
O5	0.0166 (6)	0.0161 (6)	0.0170 (6)	−0.0037 (5)	−0.0039 (5)	0.0051 (5)
C51	0.0139 (8)	0.0151 (8)	0.0152 (9)	−0.0004 (6)	0.0004 (7)	0.0049 (7)
C52	0.0203 (9)	0.0176 (9)	0.0283 (11)	−0.0007 (7)	0.0037 (8)	−0.0027 (8)
C53	0.0209 (9)	0.0258 (10)	0.0328 (12)	0.0045 (8)	0.0084 (8)	−0.0029 (9)
C54	0.0137 (9)	0.0353 (11)	0.0287 (11)	−0.0021 (8)	0.0006 (8)	0.0035 (9)
C55	0.0195 (9)	0.0303 (10)	0.0249 (11)	−0.0091 (8)	−0.0015 (8)	−0.0027 (8)
C56	0.0210 (9)	0.0200 (10)	0.0181 (9)	−0.0003 (7)	0.0018 (7)	−0.0021 (7)
C61	0.0129 (8)	0.0200 (9)	0.0303 (10)	−0.0037 (7)	0.0068 (7)	−0.0011 (8)
C62	0.0278 (11)	0.0266 (11)	0.0516 (15)	0.0058 (9)	−0.0084 (10)	−0.0058 (11)
C63	0.0339 (13)	0.0348 (14)	0.092 (2)	0.0164 (10)	−0.0059 (14)	−0.0206 (14)
C64	0.0288 (12)	0.0404 (15)	0.086 (2)	−0.0080 (10)	0.0289 (13)	−0.0346 (15)
C65	0.0664 (18)	0.0438 (15)	0.0409 (15)	−0.0146 (13)	0.0332 (14)	−0.0149 (12)
C66	0.0536 (14)	0.0298 (12)	0.0301 (12)	0.0052 (10)	0.0191 (11)	0.0008 (9)
N7	0.0203 (7)	0.0211 (8)	0.0148 (7)	−0.0020 (6)	−0.0002 (6)	−0.0011 (7)
C71	0.0267 (10)	0.0290 (11)	0.0326 (12)	−0.0049 (9)	0.0121 (9)	−0.0002 (9)
C72	0.0242 (11)	0.0432 (14)	0.0539 (16)	−0.0119 (10)	0.0011 (11)	−0.0037 (11)
C73	0.0206 (9)	0.0249 (10)	0.0199 (10)	−0.0008 (7)	0.0036 (8)	−0.0047 (8)
C74	0.0366 (12)	0.0265 (11)	0.0315 (12)	0.0037 (9)	−0.0042 (10)	−0.0043 (9)
C75	0.0289 (10)	0.0254 (10)	0.0157 (9)	−0.0017 (8)	−0.0054 (8)	0.0034 (7)
C76	0.0382 (12)	0.0299 (12)	0.0255 (11)	0.0100 (9)	−0.0098 (9)	−0.0004 (9)

### Geometric parameters (Å, °)

**Table:**

Sb—O1	2.2091 (12)	C52—H52	0.9300
Sb—O2	1.9751 (11)	C53—C54	1.381 (3)
Sb—O4	2.1204 (12)	C53—H53	0.9300
Sb—O5	1.9662 (11)	C54—C55	1.378 (3)
O1—C1	1.274 (2)	C54—H54	0.9300
C1—O11	1.236 (2)	C55—C56	1.389 (2)
C1—C2	1.550 (2)	C55—H55	0.9300
C2—O2	1.4155 (19)	C56—H56	0.9300
C2—C31	1.530 (2)	C61—C62	1.373 (3)
C2—C21	1.534 (2)	C61—C66	1.380 (3)
C21—C22	1.386 (2)	C62—C63	1.383 (3)
C21—C26	1.390 (2)	C62—H62	0.9300
C22—C23	1.393 (2)	C63—C64	1.361 (4)
C22—H22	0.9300	C63—H63	0.9300
C23—C24	1.379 (3)	C64—C65	1.375 (4)
C23—H23	0.9300	C64—H64	0.9300
C24—C25	1.385 (3)	C65—C66	1.382 (3)
C24—H24	0.9300	C65—H65	0.9300
C25—C26	1.382 (2)	C66—H66	0.9300
C25—H25	0.9300	N7—C71	1.495 (2)
C26—H26	0.9300	N7—C73	1.496 (2)
C31—C36	1.386 (2)	N7—C75	1.500 (2)
C31—C32	1.393 (2)	N7—H7	0.93 (2)
C32—C33	1.384 (2)	C71—C72	1.508 (3)
C32—H32	0.9300	C71—H71A	0.9700
C33—C34	1.388 (3)	C71—H71B	0.9700
C33—H33	0.9300	C72—H72A	0.9600
C34—C35	1.374 (3)	C72—H72B	0.9600
C34—H34	0.9300	C72—H72C	0.9600
C35—C36	1.389 (3)	C73—C74	1.505 (3)
C35—H35	0.9300	C73—H73A	0.9700
C36—H36	0.9300	C73—H73B	0.9700
O4—C4	1.295 (2)	C74—H74A	0.9600
C4—O41	1.218 (2)	C74—H74B	0.9600
C4—C5	1.557 (2)	C74—H74C	0.9600
C5—O5	1.4195 (19)	C75—C76	1.516 (3)
C5—C61	1.528 (2)	C75—H75A	0.9700
C5—C51	1.532 (2)	C75—H75B	0.9700
C51—C56	1.385 (2)	C76—H76A	0.9600
C51—C52	1.393 (2)	C76—H76B	0.9600
C52—C53	1.377 (2)	C76—H76C	0.9600
			
O2—Sb—O1	76.77 (4)	C52—C53—H53	119.8
O4—Sb—O1	147.72 (5)	C54—C53—H53	119.8
O5—Sb—O1	81.60 (5)	C55—C54—C53	119.20 (17)
O2—Sb—O4	81.84 (5)	C55—C54—H54	120.4
O5—Sb—O2	100.79 (5)	C53—C54—H54	120.4
O5—Sb—O4	78.97 (5)	C54—C55—C56	120.64 (18)
C1—O1—Sb	114.31 (11)	C54—C55—H55	119.7
O11—C1—O1	124.65 (16)	C56—C55—H55	119.7
O11—C1—C2	119.54 (15)	C51—C56—C55	120.44 (17)
O1—C1—C2	115.80 (14)	C51—C56—H56	119.8
O2—C2—C31	109.24 (13)	C55—C56—H56	119.8
O2—C2—C21	108.10 (13)	C62—C61—C66	118.57 (19)
C31—C2—C21	111.00 (13)	C62—C61—C5	123.62 (18)
O2—C2—C1	109.66 (12)	C66—C61—C5	117.80 (17)
C31—C2—C1	108.62 (13)	C61—C62—C63	120.1 (2)
C21—C2—C1	110.21 (13)	C61—C62—H62	119.9
C2—O2—Sb	118.32 (9)	C63—C62—H62	119.9
C22—C21—C26	118.83 (16)	C64—C63—C62	121.1 (2)
C22—C21—C2	122.98 (15)	C64—C63—H63	119.5
C26—C21—C2	118.05 (15)	C62—C63—H63	119.5
C21—C22—C23	120.35 (17)	C63—C64—C65	119.4 (2)
C21—C22—H22	119.8	C63—C64—H64	120.3
C23—C22—H22	119.8	C65—C64—H64	120.3
C24—C23—C22	120.49 (18)	C64—C65—C66	119.7 (3)
C24—C23—H23	119.8	C64—C65—H65	120.2
C22—C23—H23	119.8	C66—C65—H65	120.2
C23—C24—C25	119.20 (17)	C61—C66—C65	121.1 (2)
C23—C24—H24	120.4	C61—C66—H66	119.4
C25—C24—H24	120.4	C65—C66—H66	119.4
C26—C25—C24	120.56 (17)	C71—N7—C73	113.47 (15)
C26—C25—H25	119.7	C71—N7—C75	114.21 (15)
C24—C25—H25	119.7	C73—N7—C75	110.58 (14)
C25—C26—C21	120.55 (17)	C71—N7—H7	106.6 (12)
C25—C26—H26	119.7	C73—N7—H7	107.1 (12)
C21—C26—H26	119.7	C75—N7—H7	104.1 (12)
C36—C31—C32	119.05 (16)	N7—C71—C72	113.86 (17)
C36—C31—C2	120.90 (15)	N7—C71—H71A	108.8
C32—C31—C2	120.03 (15)	C72—C71—H71A	108.8
C33—C32—C31	120.62 (17)	N7—C71—H71B	108.8
C33—C32—H32	119.7	C72—C71—H71B	108.8
C31—C32—H32	119.7	H71A—C71—H71B	107.7
C32—C33—C34	120.00 (17)	C71—C72—H72A	109.5
C32—C33—H33	120.0	C71—C72—H72B	109.5
C34—C33—H33	120.0	H72A—C72—H72B	109.5
C35—C34—C33	119.42 (17)	C71—C72—H72C	109.5
C35—C34—H34	120.3	H72A—C72—H72C	109.5
C33—C34—H34	120.3	H72B—C72—H72C	109.5
C34—C35—C36	120.97 (17)	N7—C73—C74	112.90 (15)
C34—C35—H35	119.5	N7—C73—H73A	109.0
C36—C35—H35	119.5	C74—C73—H73A	109.0
C31—C36—C35	119.93 (17)	N7—C73—H73B	109.0
C31—C36—H36	120.0	C74—C73—H73B	109.0
C35—C36—H36	120.0	H73A—C73—H73B	107.8
C4—O4—Sb	116.24 (11)	C73—C74—H74A	109.5
O41—C4—O4	124.22 (17)	C73—C74—H74B	109.5
O41—C4—C5	120.70 (16)	H74A—C74—H74B	109.5
O4—C4—C5	115.08 (15)	C73—C74—H74C	109.5
O5—C5—C61	108.09 (14)	H74A—C74—H74C	109.5
O5—C5—C51	107.60 (13)	H74B—C74—H74C	109.5
C61—C5—C51	112.32 (14)	N7—C75—C76	111.77 (16)
O5—C5—C4	110.16 (14)	N7—C75—H75A	109.3
C61—C5—C4	111.56 (14)	C76—C75—H75A	109.3
C51—C5—C4	107.04 (14)	N7—C75—H75B	109.3
C5—O5—Sb	119.22 (10)	C76—C75—H75B	109.3
C56—C51—C52	118.33 (16)	H75A—C75—H75B	107.9
C56—C51—C5	123.58 (15)	C75—C76—H76A	109.5
C52—C51—C5	118.09 (15)	C75—C76—H76B	109.5
C53—C52—C51	120.98 (17)	H76A—C76—H76B	109.5
C53—C52—H52	119.5	C75—C76—H76C	109.5
C51—C52—H52	119.5	H76A—C76—H76C	109.5
C52—C53—C54	120.38 (18)	H76B—C76—H76C	109.5
			
O5—Sb—O1—C1	115.32 (12)	Sb—O4—C4—O41	178.69 (14)
O2—Sb—O1—C1	12.08 (11)	Sb—O4—C4—C5	−0.40 (18)
O4—Sb—O1—C1	61.90 (15)	O41—C4—C5—O5	−174.87 (16)
Sb—O1—C1—O11	177.04 (14)	O4—C4—C5—O5	4.3 (2)
Sb—O1—C1—C2	−2.13 (18)	O41—C4—C5—C61	65.1 (2)
O11—C1—C2—O2	167.18 (15)	O4—C4—C5—C61	−115.79 (16)
O1—C1—C2—O2	−13.6 (2)	O41—C4—C5—C51	−58.2 (2)
O11—C1—C2—C31	47.9 (2)	O4—C4—C5—C51	120.97 (16)
O1—C1—C2—C31	−132.91 (15)	C61—C5—O5—Sb	115.54 (12)
O11—C1—C2—C21	−73.93 (19)	C51—C5—O5—Sb	−122.94 (12)
O1—C1—C2—C21	105.29 (16)	C4—C5—O5—Sb	−6.58 (17)
C31—C2—O2—Sb	144.16 (10)	O2—Sb—O5—C5	−74.24 (11)
C21—C2—O2—Sb	−94.96 (13)	O4—Sb—O5—C5	5.13 (11)
C1—C2—O2—Sb	25.23 (16)	O1—Sb—O5—C5	−148.95 (11)
O5—Sb—O2—C2	−99.33 (11)	O5—C5—C51—C56	−125.51 (17)
O4—Sb—O2—C2	−176.37 (11)	C61—C5—C51—C56	−6.7 (2)
O1—Sb—O2—C2	−20.72 (10)	C4—C5—C51—C56	116.10 (19)
O2—C2—C21—C22	110.46 (18)	O5—C5—C51—C52	53.7 (2)
C31—C2—C21—C22	−129.76 (17)	C61—C5—C51—C52	172.54 (16)
C1—C2—C21—C22	−9.4 (2)	C4—C5—C51—C52	−64.69 (19)
O2—C2—C21—C26	−65.23 (18)	C56—C51—C52—C53	1.4 (3)
C31—C2—C21—C26	54.6 (2)	C5—C51—C52—C53	−177.87 (17)
C1—C2—C21—C26	174.93 (14)	C51—C52—C53—C54	−0.1 (3)
C26—C21—C22—C23	1.9 (3)	C52—C53—C54—C55	−0.8 (3)
C2—C21—C22—C23	−173.74 (16)	C53—C54—C55—C56	0.4 (3)
C21—C22—C23—C24	−1.1 (3)	C52—C51—C56—C55	−1.7 (3)
C22—C23—C24—C25	−0.2 (3)	C5—C51—C56—C55	177.48 (17)
C23—C24—C25—C26	0.7 (3)	C54—C55—C56—C51	0.8 (3)
C24—C25—C26—C21	0.2 (3)	O5—C5—C61—C62	−142.21 (17)
C22—C21—C26—C25	−1.5 (3)	C51—C5—C61—C62	99.2 (2)
C2—C21—C26—C25	174.40 (16)	C4—C5—C61—C62	−21.0 (2)
O2—C2—C31—C36	−14.4 (2)	O5—C5—C61—C66	39.1 (2)
C21—C2—C31—C36	−133.47 (17)	C51—C5—C61—C66	−79.4 (2)
C1—C2—C31—C36	105.21 (18)	C4—C5—C61—C66	160.38 (17)
O2—C2—C31—C32	167.26 (14)	C66—C61—C62—C63	−0.8 (3)
C21—C2—C31—C32	48.2 (2)	C5—C61—C62—C63	−179.45 (19)
C1—C2—C31—C32	−73.17 (19)	C61—C62—C63—C64	0.1 (4)
C36—C31—C32—C33	0.9 (3)	C62—C63—C64—C65	0.9 (4)
C2—C31—C32—C33	179.26 (16)	C63—C64—C65—C66	−1.3 (4)
C31—C32—C33—C34	0.1 (3)	C62—C61—C66—C65	0.5 (3)
C32—C33—C34—C35	−0.9 (3)	C5—C61—C66—C65	179.2 (2)
C33—C34—C35—C36	0.7 (3)	C64—C65—C66—C61	0.6 (4)
C32—C31—C36—C35	−1.1 (3)	C73—N7—C71—C72	60.8 (2)
C2—C31—C36—C35	−179.46 (16)	C75—N7—C71—C72	−67.2 (2)
C34—C35—C36—C31	0.3 (3)	C71—N7—C73—C74	56.6 (2)
O5—Sb—O4—C4	−2.44 (12)	C75—N7—C73—C74	−173.54 (16)
O2—Sb—O4—C4	100.31 (12)	C71—N7—C75—C76	−59.8 (2)
O1—Sb—O4—C4	51.60 (15)	C73—N7—C75—C76	170.79 (16)

### Hydrogen-bond geometry (Å, °)

**Table:**

*D*—H···*A*	*D*—H	H···*A*	*D*···*A*	*D*—H···*A*
N7—H7···O11	0.93 (2)	1.78 (2)	2.706 (2)	177.5 (18)
